# Left behind? A longitudinal ecological study of ‘regional deprivation amplification’ and life expectancy growth in in England (2004 to 2020)

**DOI:** 10.1016/j.healthplace.2025.103478

**Published:** 2025-05-06

**Authors:** Julija Simpson, Viviana Albani, Luke Munford, Clare Bambra

**Affiliations:** a Population Health Sciences Institute, Newcastle University, Ridley 1 Building, NE1 4LP, UK; b Health Organisation, Policy and Economics Research Team, School of Health Sciences, University of Manchester, UK

**Keywords:** Health inequalities, Health geography, Social determinants, Decomposition

## Abstract

Geographical inequalities in health are substantial and increasing in many countries. In England, there is a life expectancy gap amongst the 20 % most deprived local authorities – between those in the northern regions and those in the rest of the country. We sought to quantify the size and evolution of this gap and to investigate potential contributing factors.

We used data from official national statistics covering years 2004–2020 for the 20 % most deprived local authorities in England, divided into north and rest of England. We conducted a Blinder-Oaxaca decomposition which quantified the size of the life expectancy gap for both men and women and identified the key contributing factors drawing on ‘deprivation amplification’ concept and other theories of health inequalities.

We have found that there is a long-standing and widening gap in life expectancy between local authorities in the north and the rest of England. The gap is greater for women than for men (11.7 vs. 7.0 months on average); the widening of the gap over the past two decades has also been greater for women. Our decomposition analysis indicates that regional differences in income are the main contributor to this gap for both men and women (explaining 69 % and 44 % of the gap, respectively), with behavioural factors such as smoking having no explanatory power.

Overall, our findings suggest that providing additional income-based resources to areas lagging behind in life expectancy may be an effective way of reducing place-based health inequalities both in England and in similar regionally imbalanced economies.

## 1 Introduction

### 1.1 Background

Geographical inequalities in health are large and growing in most high-income nations, with clear regional divides in life expectancy becoming increasingly apparent in countries such as Italy, the USA, and the UK. For example, people living in the northern province of Trentino in Italy can expect to live three years longer on average than those living in the southern province of Campania (Istat, 2018). Similarly, in the southern states of the USA, adult mortality rates are 30–40 % higher than in the better performing states of the Pacific Coast, Upper Midwest and New England – a phenomenon known as the ‘southern disadvantage’ (Fenelon, 2013). In the UK, on the other hand, there is a long-standing Scottish mortality disadvantage as well as a *northern* health disadvantage, whereby the northern regions of England have worse health outcomes and shorter life expectancy (by two years on average) than regions in the rest of the country (Public Health England, 2019; McCartney et al., 2012a). Investigating such geographical patterning of health and longevity can help reveal societal, political, and environmental antecedents for health inequalities (Pearce et al,. 2015).

The most common explanation for the north-south divide is the higher levels of deprivation in the northern regions (Bambra, 2016; Woods et al., 2005; Bambra and Martin, 2024). For example, whilst the north represents 30 % of population in England, it includes 50 % of the most deprived areas (Whitehead, 2014). However, it is becoming increasingly recognised that area-level deprivation, as currently measured, is unlikely to be the only explanation. In their 2014 report Whitehead and colleagues (2014) have found that, since the early 2000s, a gap in life expectancy growth had emerged amongst the 20 % most deprived local authorities in England – between the north and those in the rest of England. By 2010, the annual life expectancy growth in the deprived areas in the north was around 6 months lower than in the deprived areas in the rest of England (Whitehead, 2014). This patterning was also observed for mortality from Covid-19 (Munford et al., 2022).

In our study, we aim to explore and empirically test the potential theory-led explanations behind the growing divide. As such, we aim to contribute to the broader body of literature on spatial health inequalities focusing on factors above and beyond area-level deprivation.

### 1.2 Health and deprivation

One of the first studies to compare health outcomes between areas with similar deprivation levels in England was the study by Phillimore and Morris in 1991 (Phillimore and Morris, 1991). This comparative study of premature mortality (under 65 years) between two similarly deprived northern English towns – Middlesbrough and Sunderland – has found that that between 1975 and 1986 premature mortality was consistently higher in Middlesbrough. The authors suggested that potential reasons for the disparity could reflect differences in provision and use of healthcare services as well as environmental differences in terms of built environment and atmospheric pollutants (although these explanations were not empirically tested). Additionally, the authors argued that past levels of health (as measured by infant mortality rates) was an unlikely explanation, given that historical infant mortality rates were higher in Sunderland. This explanation was first put forward in a study by Barker and Osmond (1986) in which they found that spatial differences in mortality in England and Wales between 1911 and 1914 were largely explained by past health conditions, such as maternal mortality.

A slightly more recent cross-sectional study by Doran et al. (2006) has concluded that life expectancy in English local authorities was associated not only with material deprivation, but with the local sociodemographic context and region where the local authority is located, with London local authorities having much higher life expectancies than their deprivation levels would predict. The potential (but untested) explanations proposed by the authors included: close proximity of London to the least deprived regions in England, higher inward migration and limitations of measures of deprivation (ibid). The findings of a protective ‘London effect’ have also been found to apply to self-rated health (Whynes, 2009), indicating that deprivation level is only a part of an explanation for spatial health inequalities in England.

The same has also been found to be true across the regions of the UK, with Scotland consistently underperforming in terms of health relative to England – i.e., ‘the Scottish mortality disadvantage’ (Collins and McCartney, 2011; McCartney et al., 2012b). More specifically, Hanlon et al. (2005) has found that between 1981 and 2001, mortality rates in Scotland were 17 % higher compared to similarly deprived areas in England and Wales, with the gap being greater for more deprived deciles and increasing over time. A dialectic review of potential explanations of this phenomenon has highlighted the importance of factors such as inadequate measurement of deprivation, high levels of overcrowding historically, and a more profound experience of deindustrialisation (Walsh et al., 2017).

The importance of political factors including poor management of deindustrialisation have also been underscored in studies exploring why Glasgow also underperforms in terms of health relative to similar post-industrial cities in England, such as Liverpool or Manchester (Walsh et al., 2010, 2017). The effects of economic and social policies, and in particular neoliberalism, in creating and maintaining health divides is also evident in countries both across Europe and the USA (Taulbut et al., 2014; Wami et al., 2021).

Taken together, this evidence underscores the importance of historical, political and other related factors for spatial health inequalities, above and beyond area-deprivation levels alone. However, outside of the Scottish work, there has been very little examination of the potential explanations - behind why the health effects of deprivation vary by place (Walsh et al., 2017). Indeed, factors relating to the north-south divide in deprivation, remain both under-theorised and empirically untested. Our paper aims to fill this gap.

### 1.3 Deprivation amplification hypothesis

The conceptual framework for our analysis is the ‘deprivation amplification’ hypothesis (Munford et al., 2022). This hypothesis asserts that local context can compound individual-level disadvantage (i.e., the health of deprived people living in more deprived areas tends be worse than for deprived people living in more affluent areas) (Macintyre, 2007). By extension, the health effects of local area-level deprivation can be compounded - or ‘amplified’ – by the wider geographical context (i.e. deprived areas located within deprived regions fare worse than deprived areas within more affluent regions) – a process known as *regional deprivation amplification* (Munford et al., 2022)

The theory of ‘deprivation amplification’ draws on the wider health geography literature – particularly on the context-composition-relational debate (Cummins et al., 2007). The compositional view argues that it is the characteristics of who lives in a place that determines area health outcomes (Bambra et al., 2019). Such characteristics comprise socio-demographic characteristics (age, sex, ethnicity), health-related practices/behaviours, and socio-economic profile of people living within a particular place. The contextual approach, on the other hand, highlights that it is the economic, social, and physical environments of a place that matter for residents’ health. Contextual factors include unemployment and wage rates; public and private service provision; as well as noise, air pollution, and green spaces. Both context and composition interact relationally, with context influencing compositional characteristics (Macintyre et al., 2002).

The contextual and compositional characteristics of place are also influenced by macro political and economic factors - the ‘political economy of health’, whereby spatial patterns of health and disease are shaped by the structures, values and priorities of the wider political, social and economic system (Krieger, 2003; Schrecker and Bambra, 2025). These in turn impact the social, economic and physical environment and the social and spatial distribution of salutogenic and pathogenic factors at composition and contextual scales (Bambra, 2016). For instance, evidence shows that the cuts to public expenditure under the ‘austerity’ policies of the 2010s were spatially uneven – with areas in the north experiencing significantly greater losses in local authority and welfare spending than those in the rest of England (Beatty and Fothergill, 2018; Gray and Barford, 2018; Ogden, 2024) – potentially resulting in disproportionate effects on the disposable incomes and access to services in the north, amongst other impacts.

The aim of this paper is to examine if there is regional deprivation amplification in life expectancy in England and to explore the factors contributing to this. Specifically, we (1) estimate the size of the difference in life expectancy amongst the 20 % most deprived Local Authorities in England, comparing those located in the north with those located in the rest of England from 2004 to 2020; and (2), examine how compositional, contextual, and political economy factors contribute to the evolution of the differences. As such, our paper contributes to the literature empirically – through providing new estimates of regional health inequalities in England, methodologically – by applying the decomposition technique from labour economics into health geography, and theoretically – by testing theories of health and place.

## 2 Methods

### 2.1 Sample

The sample in our study includes the 20 % most deprived local authorities (LAs) in England throughout the period of our study – i.e., deprived according to the 2010, 2015 and 2019 Index of Multiple Deprivation (IMD), resulting in a total of 51 LAs (30 in the north and 21 in the rest of England (MHCLG, 2024). The IMDs for years 2010, 2015, 2019 are comparable and the recommended indicators of deprivation for our analysis period, with IMD (2010) capturing deprivation for years 2004–2010; IMD (2015) – for years 2011–2015; and IMD (2019) – for years 2016 onwards (Department of Health and Social Care, 2024).

In line with recent literature on regional health inequalities in England (Munford et al., 2022; Bambra et al., 2023), we define the north as the North East, North West and Yorkshire and the Humber, with the rest of England comprising the remaining six regions (South East, South West, London, East Midlands, West Midlands, and East of England).

The full list of included LAs and sample selection process is presented in Appendix A. Given data availability, our analysis timeframe is 2004–2020, resulting in the total sample size available of 867 observations. From these, we had to exclude 31 observations due to missing values, giving us the final sample of 836 observations for the main analysis.

### 2.2 Outcome variable

Our primary outcome variable was male and female life expectancy at birth between 2004 and 2020 by LA in England. These measures were provided by Office for National Statistics (ONS, 2024a) and aggregated over rolling three-year intervals to account for annual fluctuations in mortality in relatively small populations. In our analyses of annual trends, we took the middle calendar year as the reference year.

### 2.3 Explanatory variables

Our variable selection process was informed by theories of geographical inequalities in health and by data availability. We conceptualised variables into one of ‘Context’, ‘Composition’, or ‘Political economy’ groups based on whether they were individual socio-demographic characteristics, collective local environmental level factors or macro-level political and economic elements respectively.

Included variables relating to ‘composition’ theory are: ethnicity (as a social factor, proxied by the ‘White’ proportion of the population); education (proportion of adults with Level 3 qual. (A-levels) or above)); international migration (proxied by the proportion of non-British born people); median gross disposable annual household income (in £s and adjusted for inflation using the Consumer Price Index) – an estimate of the amount of money each individual within a household has available for spending or saving after they have paid taxes and received any direct benefits and is classed as an ‘indicator of material welfare by the Office for National Statistics (ONS, 2021). It should be noted that, given that age is already adjusted for in calculation of our outcome (life expectancy), we do not additionally include it in our analyses.

The ‘context’ variables are local authority unemployment and inactivity rates; job density (measured by the number of jobs available per person); local occupational profile (the proportion of the labour force working in elementary occupations); industrial sector (proportion of all employed in manufacturing industry); wage (median hourly wage); productivity (gross-value added (GVA) per hour worked); economic growth (gross national product (GDP)) – with the latter three variables all adjusted for inflation.

The ‘political economy’ variables included are: welfare benefit losses per working-age individual in upper-tier LAs (capturing cumulative benefit losses from welfare reforms between 2010 and 2015), were obtained from Seaman et al. (2024) and local authority spending per capita, adjusted for inflation and obtained from the Place-based Longitudinal Data Resource (PLDR, 2021).

It should be noted that ‘composition’ theory-related variables which were of potential importance for the main analysis but had limited data (i.e., not available before 2012) were included as part of our sensitivity analyses: i.e., internal migration and smoking. Internal net migration was measured by subtracting outflows from LA from inflows into LA and divided by the LA’s population size, whereby the inflows and outflows were constructed from new GP registration data. These data were obtained from ONS (ONS, 2024b). The variable indicating smoking prevalence (our proxy for health behaviour) includes the proportion of adults who state they currently smoke cigarettes and was sourced from the APS (ONS, 2024c).

The full list of available variables relating to composition, context, and political economy explanations and their sources are presented in Table 1. Given the potential heterogeneity in terms of exposure to deprivation within our sample, in our decomposition analysis we have additionally included the value of the deprivation rank for each local authority as a control. In our descriptive statistics, we have also provided summaries of each IMD domain. These variables were not included in our analysis because they are only available at two time points (for IMDs, 2015 and 2019) and given that we have already controlled for local authority deprivation rank based on weighted average of these domains. Details relating to description of each domain can be found in IMD technical report (McLeanan Dn et al., 2019).

### 2.4 Statistical analysis

To investigate and mitigate against potential multi-collinearity issues amongst some of the variables, we first estimated pooled Ordinary Least Squares (OLS) regression models including all the above variables, separately for male and female life expectancies as outcomes, and obtained variable inflation factors (VIFs) related to all explanatory variables post-estimation.

The variables with the greatest VIFs included wages, proportion of non-British nationals, ethnicity, and GVA (VIFs>10, indicating a high likelihood of multi-collinearity problems (Vittinghoff et al., 2005)). We have therefore removed these variables from our main analysis one at a time until all variables had VIFs below 10. This has meant that all of the aforementioned variables were removed from the main analysis (except ethnicity as its VIF had gone down after removing the proportion of non-British). A correlation matrix of all original variables is presented in Appendix B.

#### 2.4.1 Decomposition analysis

To understand which factors are contributing to the observed gap in life expectancy between the most deprived local authorities in the north vs. rest of England, we used a Blinder-Oaxaca Decomposition approach. This approach was originally developed in the labour economics literature to understand the potential factors contributing to the wage gap between men and women and how much of it is explained by observed and unobserved characteristics (Blinder, 1973; Oaxaca, 1973). Since its development, the method has been increasingly used in health inequalities research (Allen et al., 2022; Brown et al., 2024; Rahimi et al., 2021).

Following this literature, we estimated a two-fold Blinder-Oaxaca decomposition to explore the relative contributions of the factors related to alternative explanations of regional health inequalities in the observed gap in life expectancy between the deprived north and the rest of England (i.e., the contributions of Context, Composition and Political Economy related variables), whereby the difference in life expectancy (LE) can be decomposed as:

(1) $${\bar{LE}}_{Rest}-{\bar{LE}}_{North}=\left({\bar{X}}_{Rest}-{\bar{X}}_{North}\right){\bar{\beta }}_{Rest}+{\bar{X}}_{North}\left({\bar{\beta }}_{Rest}-{\bar{\beta }}_{North}\right)$$

The left-hand side represents the mean observed difference in life expectancy amongst the 20 % most deprived Local Authorities between the north and rest of England. The first term on the right-hand side captures the part of the difference attributable to differences in observed characteristics between the two region groups (‘explained’ or ‘endowment’ part), and the second term indicates the part of the difference that is due to unobserved characteristics (‘coefficient’ or ‘unexplained’ part).

*X* includes factors related to composition, context, and political economy explanations for health inequalities outlined in Table 1, except for variables including the proportion of non-British nationals, ethnicity, and GVA due to high multi-collinearity, as explained above.

The basic Blinder-Oaxaca decomposition approach selects one group as the comparison group (in our case Rest of England) and one group as the reference group (north of England). When estimating the model, switching the reference group may change the results. Following the suggestion from Oaxaca and Ransom (1994) and (Neumark, 2004), we used the estimates from a pooled sample of the two models, where each model used the alternative group as the reference group (Oaxaca and Ransom, 1994; Neumark, 2004; Jann, 2008).

### 2.5 Sensitivity analyses

To explore the robustness of our results to the addition of further ‘Composition’ variables which had limited data (from 2012) – i.e., smoking and internal migration, we have included these variables (separately and together) to our main models.

In addition, we have explored whether differences in life expectancy between north and rest of England are driven by London – a hypothesis supported by prior literature on north-south divide. More specifically, Minton and McCartney (2018) point out that north-south health disparities in young adults (25–44 years old) are largely differences between London and not London (Kontopantelis et al., 2018), echoing the findings of studies by Doran et al. (2006) and Whynes (2009).

## 3 Results

### 3.1 Descriptive characteristics

As illustrated in Table 2, there are significant differences between deprived local authorities in the north and the rest of England in terms of most of our included characteristics. First, there are significant differences in life expectancy, with women in the north living around one year (or 12 months) less than those in the rest of England. For men, the gap is smaller – equal to around 0.6 years (or 7 months).

The deprived local authorities in the north have a slightly less educated population; a considerably greater proportion of White ethnicity individuals; lower job density; a greater proportion employed in the Manufacturing industry; lower GVA; greater welfare benefit losses and lower on average local authority service expenditure per capita. Crucially, deprived local authorities in the north have significantly lower income levels than those in the rest of England (£13,630 vs. £15,715).

Looking at deprivation ranks, local authorities in the north are slightly more deprived on average (full distributions of ranks for north and rest of England are illustrated in Appendix C). To investigate the extent of deprivation by IMD domain, we used the readily available measure of the Proportion of Lower Super Output Areas ((LSOAs) smaller level geographical units) within each LA in the most deprived 10 % nationally. We can see that local authorities in the north have a greater proportion of LSOAs that are amongst 10 % most Income deprived nationally, equal to 27 % (vs. 21 % in the rest of England). For the domains of Employment, Education and Health, the northern LAs also have a greater share of deprived LSOAs. In contrast, LAs in the rest of England have a greater proportion of deprived LSOAs in terms of Barriers to Housing and Services, whereas the domains of Crime and Living Environment have similar shares of deprived LSOAs in the north and rest of England.

### 3.2 Graphical trends in life expectancy

**Fig. 1:**
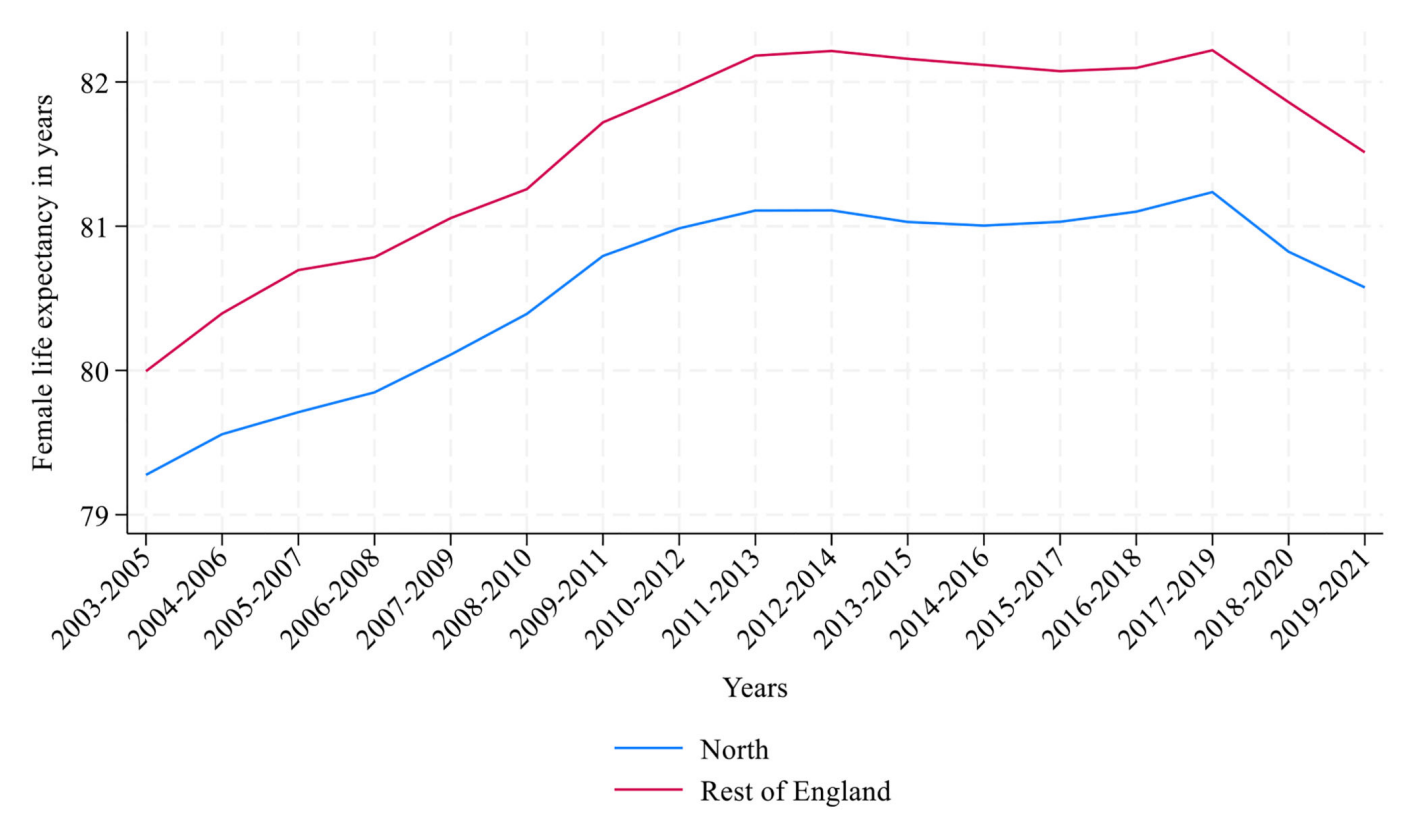
Female life expectancy amongst the most deprived 20 % of national local authorities in the North and Rest of England (in years).

**Fig. 2:**
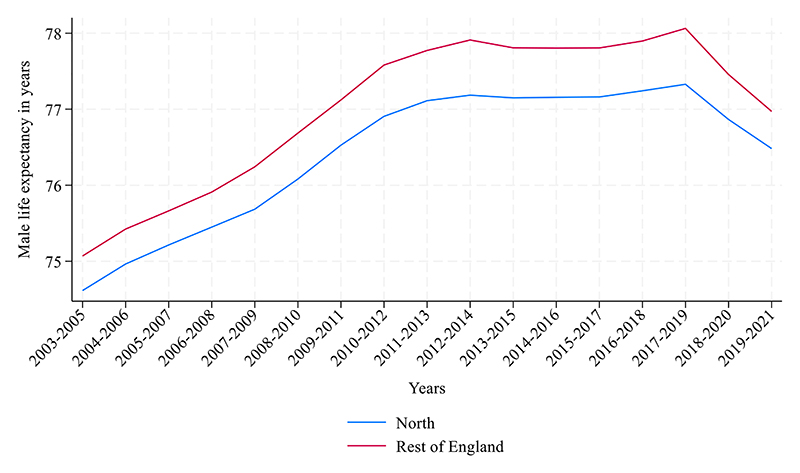
Male life expectancy amongst the most deprived 20 % of national local authorities in the North and Rest of England (in years).

Figs. 1 and 2 illustrate the long-standing and widening divides in life expectancy amongst the most deprived 20 % of local authorities between the north and rest of England for both women (Fig. 1) and men (Fig. 2), with a greater divide for women. Since 2004, the gap for women increased from 0.72 years to 0.94 years in 2020 (from 8.64 to 11.28 months), indicating a 31 % increase. For men, the increase was smaller – from 0.46 to 0.49 years or from 5.5 to 5.9 months (7 % increase).

Graphs for all the explanatory variables can be found in Appendix D.

### 3.3 Decomposition results

Table 3 presents the results of the Blinder-Oaxaca decomposition analysis for women and men respectively.

The results show that there is a statistically significant difference in life expectancy amongst the most deprived 20 % of national local authorities between LAs in the north and rest of England across the period of our study (2004–2020) for both men and women, equal to 7.01 and 11.70 months respectively (on average).

For women, differences in observed characteristics, constitute 41 % of this difference (4.77 months), with 59 % unexplained. The ‘unexplained’ component indicates the proportion of regional inequality that would remain even if the north had the same levels of the included variables as the rest of England (Sen, 2014).

The greatest contributor to the gap from the ‘explained’ (or ‘endowments’) part is median household income. More specifically, the results suggest that if the north had the same levels of income as the rest of England, the gap in life expectancy amongst the most deprived 20 % of national local authorities would be reduced by approximately 5.15 months (44 % of the total gap). Most other variables also contribute to the gap (positively or negatively, whereby a negative coefficient means that the current levels of the variable work to reduce the gap), with main contributors being ethnicity (0.92 months or 8 % of the gap), unemployment (0.82 months or 7 %), and education (0.35 months or 3 %). Local authority service expenditure, on the other hand, has a negative coefficient (−2.77 months), suggesting that it has a gap-reducing effect.

For men, differences in observed characteristics explain 59 % of the overall difference (4.17 out of 7.01 months). Like for women, the greatest contributor from the ‘explained’ part of the gap is median income (explaining 69 % of the gap). The remaining contributors include unemployment and industrial sector composition (12 % each), followed by education (8 %) and welfare benefit losses (6 %). These figures add up to over a 100 % because the contributions of several other variables, including job density and local authority expenditure are negative (gapreducing).

It should be noted that detailed results for the ‘unexplained’ component do not have a meaningful interpretation as they can be driven by factors including unobserved characteristics not included in the model, measurement error, or discrimination (Sen, 2014).

Given the importance of both income and welfare benefit losses in explaining the life expectancy gap between the north and the rest of England for men, we have further investigated the relationship between these two variables. Our supplementary analysis results in Appendix E suggest that median household income and welfare benefit losses are indeed negatively associated, suggesting that welfare benefit losses may in part explain the income effect. A simple segmented regression analysis also indicates that there was a significant change in household income trends following the onset of austerity in 2010, with north lagging yet further behind the rest of England in terms of median household income, indicating the interdependence between the two variables (though it is not perfect or very high collinearity, with correlation co-efficient equal to −0.12).

### 3.4 Sensitivity analysis results

Including variables for internal migration have not changed our main conclusion that income was the main contributor to the life expectancy gap for both men and women. Neither internal migration nor smoking had statistically significant contributions to the life expectancy gap, as illustrated in Appendix F.

Investigation of differences in life expectancy by region in Appendix G does not show a consistent pattern with London being ‘an outlier’ in terms of life expectancy for either men or women (instead, LAs in South West have consistently higher life expectancy), suggesting that this hypothesis may not hold true for our sample.

## 4 Discussion

### 4.1 Summary and contextualisation of key findings

Our findings suggest that there is a long-standing and widening gap in life expectancy amongst the most deprived 20 % of national local authorities between those in the north and those in the rest of England. The gap is greater for women than for men (11.7 vs. 7.0 months on average throughout the study period). The widening of the gap over the past two decades has also been greater for women – by 31 % vs. 7 % for men. Our decomposition analysis indicates income is the main contributor to this gap for both men and women, with most other factors having only small contributions.

The finding of a persistent and widening gap in life expectancy between deprived areas in the north and deprived areas in the rest of England provides support to the theory of deprivation amplification (Macintyre, 2007). It suggests that it is not just the immediate neighbourhood that matters for population health but also wider regional context. Our study contributes to the growing evidence base applying this theory and supporting its use in research of geographical health inequalities (Bambra, 2016; Munford et al., 2022).

Additionally, our study provides evidence for the importance of income in explaining place-based health inequalities. For example, our results are in line with a previous study showing that it is income-related deprivation in the north that is a contributor to its health disadvantage (Woods et al., 2005). Broader literature on health inequalities similarly suggests that policies that increase income (including from welfare benefits) can help reduce health inequities (Simpson et al., 2021, 2024; Richardson et al., 2020). Welfare benefit or income losses, on the other hand, tend to have the opposite effect (Simpson et al., 2021). Indeed, our decomposition results indicate welfare benefit losses as another factor contributing to the life expectancy gap amongst the most deprived 20 % of national local authorities between the north and rest of England, although only for men. Behavioural factors such as smoking, on the other hand, did not contribute to explaining life expectancy gaps for either men or women, in contrast to the common public health messaging driven by behaviouralist explanations of health inequalities.

The above findings also highlight the relational nature of our variables of interest. Namely, our further analyses suggest that median household income and welfare benefit losses are negatively related and that there was a significant change in household income trends in the north vs. rest of England following the onset of austerity in 2010 – with north lagging further behind in terms of median household income. This underscores the importance of considering the interdependence of different explanations to spatial health inequalities (e.g., population composition and political economy in this case) which may help generate policy-relevant insights for reducing health inequalities.

Our finding that the effect of local authority service expenditure, was gap-reducing went contrary to our expectations – given on average greater LA service cuts in the north (as well as in several London LAs) (Ogden, 2018). This could potentially be explained by the fact that, amongst the subset of 20 % most deprived LAs, over time local authorities in the rest of England (primarily London LAs) had greater service expenditure losses than those in the north, leading to the total expenditure levels per capita to converge towards the end of the study period, as illustrated in Appendix D.

We have additionally found that, alongside income, ethnicity and industrial sector composition also contribute to explaining the life expectancy gaps between the north and rest of England for women and men respectively. With regards to ethnicity, these findings are consistent with prior literature examining differences between London and rest of England as well as the Scottish research – all suggesting the ‘healthy migrant effect’ as a protective factor (Whynes, 2009; Walsh et al., 2017). In addition, these findings are in line with the broader ethnic density literature, whereby communities with greater ethnic density tend to have better social support networks and are more resilient to economic deprivation as a result (Cairns et al., 2012). In terms of industrial sector composition, the higher proportion of manufacturing sector employment in the north and its contribution to the life expectancy gap for men are likely indicative of the importance of industry in the region as well as the more pronounced effects of de-industrialisation – a historical factor associated with increased vulnerability of local areas to exposures such as economic and political changes both in the UK and internationally (Walsh et al., 2017; Taulbut et al., 2014; Murray et al., 2006). Again, the latter has been highlighted in relation to Scotland (Collins and McCartney, 2011).

Finally, our findings support the increasing calls for improved measurement of area-level deprivation (Whynes, 2009; Walsh et al., 2017). The regional differences within the 20 % most deprived local authorities suggest that future research should investigate how to improve the measurement of deprivation (e.g., by accounting for local historical context and increased vulnerability to contractionary policies like austerity) and to thus better inform resource allocation as well as policy approaches to reducing health inequalities. A recent example of such a strategy includes the Core20PLUS5 – a national approach to inform action to reduce healthcare inequalities in England at both national and system level. The approach defines a target population – the ‘Core20’ (the most deprived 20 % of the national population) and identifies ‘5’ focus clinical areas requiring accelerated improvement including maternity, severe mental illness, chronic respiratory disease, early cancer diagnosis and hypertension (see NHS England (NHS England, 2025) for more detail).

### 4.2 Strengths and limitations

One of the main strengths of our study is that our decomposition analysis has not only quantified life expectancy gaps amongst deprived local authorities between those in the north and those in the rest of England but has also provided insights into the key drivers of health inequalities, enabling us to provide practical and policy-relevant recommendations for reducing health inequalities. Additionally, our variable selection process was theory-led, allowing us to select a comprehensive list of variables likely contributing to place-based health inequalities, over a time period spanning nearly two decades (2004–2020).

However, the findings of our study should be interpreted in light of its limitations. First, given data availability, all our analysis variables were at local authority level, potentially masking inequalities within each local authority. Future research using more granular (e.g., neighbourhood level data could provide a richer insight into the drivers of the North-South divide, however, it is not available at the time of this study. Relatedly, future research using more granular data could also help elucidate the factors underlying the differences in life expectancy gaps for men and women. While our findings suggest that income was the main contributor to the gap for both men and women, much of the overall gap for women remains unexplained (59 %).

Another limitation of our study is that using aggregate-level data means that our results may be prone to ecological fallacy (i.e., that population-level findings might not represent individual-level ones). However, arguably, both our exposures (variables relating to population composition, context, and political economy) and outcomes (average life expectancy) are population-level ones, not necessarily reducible to purely individual-level characteristics (Stokes et al., 2022).

Additionally, the relational and potentially endogenous nature of most variables means that we cannot interpret our results as causal. However, our results do provide an indication of an independent effect of each factor, whilst holding other observed characteristics constant, which can serve as a useful and policy-relevant indicator of the relative importance of each variable.

Relatedly, given the relatively large number of exposure measures explored in our study, for ease of interpretation and to reduce the potential issues of multi-collinearity, we relied on binary indicators of categorical characteristics (e.g., ethnicity). Future research using more detailed exposure measures may help further illuminate the factors contributing to the north-south divide.

In relation to our political economy measures (local authority spending and welfare benefit losses), unfortunately, the availability of variables and data to quantify political economy factors within a single country (especially one as centralised as England) is far more limited than when comparing between countries. The majority of political economy of health research to date has therefore been cross-national in nature – where there is more heterogeneity in terms of health and welfare systems (for example see Schrecker and Bambra (2025)). So, whilst our analysis is limited by data availability – it also attempts to operationalise the political economy approach within one territory.

Additionally, our definition of ‘deprived’ was based on overall IMD scores (which include ‘health domain’) which could therefore underestimate the true health differences between the north and rest of England. However, prior evidence suggests that the size of this bias is likely to be negligible (Bradford et al., 2023; McCartney et al., 2023).

Finally, even within the 20 % of most deprived LAs, there were significant differences between the north and rest of England in terms of area-level socio-economic characteristics, particularly, income. Future research using more granular deprivation definitions and smaller geographical units might help further elucidate our understanding on regional differences in life expectancy between the north and rest of England.

## Conclusion

In conclusion, we have found that, between 2004 and 2020 there has been a long-standing and widening gap in life expectancy between the 20 % most deprived local authorities in the north vs. in the rest of England. The diverging life expectancies between the two sets of areas within the same deprivation quintile indicates the presence of regional deprivation amplification, suggesting that life expectancy can be influenced by factors beyond the immediate individual- and neighbourhood-level environments, such as those operating at regional levels. In particular, we have found household income to be amongst the main contributors to the growing inequality in life expectancy between the north and rest of England for both men and women. Our research highlights the importance of providing additional income-based resources to deprived areas in deprived regions – a regional form of pro-portionate universalism – to reduce the long-standing and growing health divides across the country. Ultimately, this suggests that health inequalities policies need to be regional as well as local (Bambra and Martin, 2024).

## Supplementary Material

**Supplementary data**

Supplementary data to this article can be found online at https://doi.org/10.1016/j.healthplace.2025.103478.

## Figures and Tables

**Table 1:** Summary and explanation of all selected exposure variables.

Variable	Definition	Source
Ethnicity	Percentage of adults of White ethnicity	APS (ONS, 2024c)
Education	Percentage of adults with Level 3 qual. (A-levels) or above	APS (ONS, 2024c)
Nationality (proxy for international migration)	Percentage of non-British nationals	ONS (ONS, 2024d)
Income	Median gross disposable household annual income (in £s)	ONS (ONS, 2024e)
Smoking	Proportion of adults self-reporting as cigarette smokers	ONS (ONS, 2024f)
Internal migration	Net internal migration flows per capita	ONS (ONS, 2024b)
Unemployment rate	Model-based estimate of the unemployment rate among those aged 16-64	ONS (ONS, 2024f)
Inactivity rate	Percentage of individuals aged 16-64 years who were economically inactive	ONS (ONS, 2024f)
Job density	Number of jobs per person aged 16-64	ONS (ONS, 2024g)
Occupation composition	Percentage of all employed individuals who work in elementary occupations	APS (ONS, 2024c)
Industrial sector composition	Percentage of all employed individuals who work in the manufacturing industry	APS (ONS, 2024c)
Wage	Median hourly wage	ONS (ONS, 2024h)
Productivity (GVA)	Gross value added per hour worked	ONS (ONS, 2024f)
Economy size (GDP)	Gross domestic product	ONS (ONS, 2024f)
Local Authority total service expenditure	Local authority expenditure (total services, net expenditure in £s per capita)	PLDR (PLDR, 2021)
Welfare benefit losses	Total losses up to 2015 reforms (£100s per capita)	Seaman et al. (Seaman et al., 2024)

**Table 2:** Descriptive characteristics.

	North	Rest of England	Test
Life expectancy in years (women)	80.57 (0.92)	81.55 (1.08)	<0.001
Life expectancy (men)	76.42 (1.25)	77.01 (1.24)	<0.001
Proportion (prop.) with A level+	0.44 (0.07)	0.47 (0.12)	<0.001
Prop. White	0.91 (0.08)	0.75 (0.19)	<0.001
Median household income	13,630.09 (1928.60)	15,715.29 (5038.50)	<0.001
Unemployment rate	0.06 (0.02)	0.06 (0.02)	<0.001
Inactivity rate	26.72 (3.21)	26.84 (4.39)	0.829
Job density	0.72 (0.14)	0.75 (0.25)	<0.001
Prop. Manufacturing	0.09 (0.03)	0.06 (0.04)	<0.001
Prop. Elementary occupation	0.13 (0.02)	0.13 (0.04)	0.068
GVA	26.24 (4.24)	28.69 (10.05)	<0.001
Welfare benefit loss (£)	570.25 (96.53)	534.70 (81.47)	<0.001
LA expenditure (£)	1225.61 (192.61)	1356.27 (412.16)	<0.001
Deprivation rank*	41.41 (16.54)	36.97 (16.69)	<0.001
Proportion of LSOAs in most deprived 10 % nationally by IMD domain**			
Income	0.27 (0.09)	0.21 (0.09)	<0.001
Employment	0.30 (0.09)	0.17 (0.11)	<0.001
Education	0.23 (0.09)	0.16 (0.13)	<0.001
Health	0.38 (0.14)	0.13 (0.11)	<0.001
Crime	0.20 (0.12)	0.21 (0.14)	0.185
Barriers to housing & services	0.01 (0.02)	0.20 (0.28)	<0.001
Observations	527 (60.8 %)	340 (39.2 %)	

Table note: means with standard deviations in parentheses. ‘Test’ refers to test for equality between groups using linear regressions for continuous variables and Pearson χ 2 tests for factor variables. *Average deprivation rank of IMD scores, reverse coded so that higher levels represent more deprivation; **Given absent domain for IMD (2010), IMD (2015) domain measures are assigned in years 2004–2015; IMD 2019 domain measures are assigned in years 2016–2020.

**Table 3:** Blinder-Oaxaca decomposition results for females and males.

		Female				Male		
Life expectancy (in months)		Coefficient	SE	P-value		Coefficient	SE	P-value
Differential								
North		966.82	0.49	<0.01		916.95	0.67	<0.01
Rest of England		978.52	0.73	<0.01		923.96	0.84	<0.01
Gap between Rest of England and North		11.70	0.87	<0.01		7.01	1.07	<0.01
Explained		4.77	0.88	<0.01		4.17	1.10	<0.01
Unexplained		6.93	0.77	<0.01		2.84	0.80	<0.00
**Explained**								
Composition factors:								
Education (%A-levels+)		0.35	0.16	0.026		0.57	0.23	<0.01
Ethnicity (%White)		0.92	0.40	0.023		0.06	0.48	0.90
Household income (median in £)		5.15	0.77	<0.01		4.84	0.75	<0.01
Context factors:								
Unemployment (%)		0.82	0.24	<0.01		0.88	0.26	<0.01
Inactivity (%)		0.03	0.09	0.76		0.03	0.26	0.76
Job density		− 0.59	0.27	0.028		− 0.90	0.09	0.03
Industrial comp (%Manuf.)		0.45	0.25	0.079		0.88	0.32	<0.01
Occupation comp (%Elementary)		− 0.01	0.04	0.704		0.08	0.06	0.203
GVA		− 0.51	0.16	0.002		− 0.11	0.06	0.33
Political economy factors:								
Local authority expenditure (£)		− 2.77	0.49	<0.01		− 4.19	0.19	<0.01
Welfare benefit losses (£)		0.13	0.12	0.297		0.42	0.20	0.03
IMD rank of average deprivation score		0.77	0.23	<0.01		1.01	0.73	<0.01
**Unexplained**								
Composition factors:								
Education (%A-levels+)		− 1.81	4.61	0.99		6.83	5.60	0.2
Ethnicity (%White)		4.82	4.87	0.62		23.49	5.67	0.000
Income (median household in £)		− 1.62	4.75	0.12		− 18.09	6.09	0.003
Context factors:								
Unemployment (%)		− 2.41	1.58	0.53		− 4.96	1.87	0.008
Inactivity (%)		4.56	4.61	0.60		− 0.63	5.14	0.902
Job density		− 0.45	2.68	<0.01		7.75	3.00	0.010
Industrial comp (%Man)		− 5.94	1.64	<0.01		− 6.54	1.89	0.001
Occupation comp (%Elementary)		2.49	2.57	0.05		4.33	2.83	0.127
GVA		1.11	3.36	0.72		− 1.76	4.09	0.666
Political economy factors:								
Local authority expenditure (£100s)		− 2.18	3.32	0.50		− 8.68	4.21	0.039
Welfare benefit losses (£100s)		15.52	4.11	<0.01		26.86	4.87	0.000
IMD rank of average deprivation score		− 4.41	1.02	<0.01		− 3.61	1.18	0.002
Observations		836				836		

## Data Availability

Data used in this study are publicly available.
